# Commensal-Induced Regulatory T Cells Mediate Protection against Pathogen-Stimulated NF-κB Activation

**DOI:** 10.1371/journal.ppat.1000112

**Published:** 2008-08-01

**Authors:** Caitlin O'Mahony, Paul Scully, David O'Mahony, Sharon Murphy, Frances O'Brien, Anne Lyons, Graham Sherlock, John MacSharry, Barry Kiely, Fergus Shanahan, Liam O'Mahony

**Affiliations:** 1 Alimentary Pharmabiotic Centre, University College Cork, Cork, Ireland; 2 Alimentary Health Ltd., University College Cork, Cork, Ireland; Massachusetts General Hospital, United States of America

## Abstract

Host defence against infection requires a range of innate and adaptive immune responses that may lead to tissue damage. Such immune-mediated pathologies can be controlled with appropriate T regulatory (Treg) activity. The aim of the present study was to determine the influence of gut microbiota composition on Treg cellular activity and NF-κB activation associated with infection. Mice consumed the commensal microbe *Bifidobacterium infantis* 35624 followed by infection with *Salmonella typhimurium* or injection with LPS. *In vivo* NF-κB activation was quantified using biophotonic imaging. CD4^+^CD25^+^Foxp3^+^ T cell phenotypes and cytokine levels were assessed using flow cytometry while CD4^+^ T cells were isolated using magnetic beads for adoptive transfer to naïve animals. *In vivo* imaging revealed profound inhibition of infection and LPS induced NF-κB activity that preceded a reduction in *S. typhimurium* numbers and murine sickness behaviour scores in *B. infantis*–fed mice. In addition, pro-inflammatory cytokine secretion, T cell proliferation, and dendritic cell co-stimulatory molecule expression were significantly reduced. In contrast, CD4^+^CD25^+^Foxp3^+^ T cell numbers were significantly increased in the mucosa and spleen of mice fed *B. infantis*. Adoptive transfer of CD4^+^CD25^+^ T cells transferred the NF-κB inhibitory activity. Consumption of a single commensal micro-organism drives the generation and function of Treg cells which control excessive NF-κB activation *in vivo*. These cellular interactions provide the basis for a more complete understanding of the commensal-host-pathogen trilogue that contribute to host homeostatic mechanisms underpinning protection against aberrant activation of the innate immune system in response to a translocating pathogen or systemic LPS.

## Author Summary

The normal response to infection is rapid and effective clearance of pathogenic microbes. However, this immune response may occasionally cause collateral inflammatory damage to host tissue and in severe cases, such as systemic sepsis, results in organ failure. Various cellular mechanisms, including regulatory T cells, protect against aggressive immune responses. However, environmental agents which promote regulatory T cells are not well understood. We and others have previously shown that non-pathogenic or commensal micro-organisms can protect the host from aberrant pro-inflammatory activity within the gut, but the influence of these microbes on regulatory T cells in the context of systemic infection has not been examined. In this study, we demonstrate that consumption of a single commensal bacterium induces regulatory T cells *in vivo* which protect the host from pathogen-induced inflammatory responses by limiting activation of the pro-inflammatory transcription factor NF-κB via the toll-like receptor 4 (TLR-4) pathway. This report conclusively demonstrates a cellular and molecular basis for the commensal-host-pathogen trilogue resulting in enhanced protection from systemic infection whilst limiting pro-inflammatory damage mediated by activation of the innate immune system.

## Introduction

Mechanisms of host protection by commensal organisms against infection vary depending on the nature of the pathogenic challenge. They range from mutual competition for nutrients and for microbial niches, to the production of specific anti-microbial peptides [1],[2]. In addition, there is emerging evidence for engagement of host immune responses by commensal organisms [3],[4].

The host response to infection is characterised by innate and acquired cellular and humoral immune reactions, designed to limit spread of the offending organism and to restore organ homeostasis. However, to limit the aggressiveness of collateral damage to host tissues, a range of regulatory constraints may be activated. Regulatory T cells (Treg) serve one such mechanism [5]. These are derived from the thymus but may also be induced in peripheral organs, including the gut mucosa [6],[7]. CD103^+^ dendritic cells within the mucosa are largely responsible for the conversion of Tregs which is a TGF-β and retinoic acid dependent process [8],[9]. The gastrointestinal specific environmental factors that contribute to dendritic cell conversion of Tregs is likely due in part to the presence of large numbers of commensal microbes. For example, encounter with specific experimental microbes within the murine gut, has been shown to drive the development of mucosal Tregs which is associated with attenuation of inflammation in a murine model of colitis [10]. However, it is unclear whether enhancement of Treg activity by commensal organisms contributes to protection of the host from mucosal damage and systemic inflammation associated with infection by invasive pathogens.

Innate pro-inflammatory signalling in response to microbial exposure is mediated by activation of transcription factors, such as NF-κB, resulting in the transcription of a battery of effector molecules contributing to host defence and inflammation [11]. A number of bacterial products have been identified which directly block activation of the NF-κB pathway in epithelial cells via a range of novel mechanisms including the blockade of Iκ-B poly-ubiquination by non-pathogenic Salmonella strains [12] or the enhancement of NF-κB export from the nucleus by *Bacteroides thetaiotaomicron* [13]. However, these molecular events are likely to be restricted to the gastrointestinal mucosa where direct interactions with these bacterial organisms takes place. The cellular mechanisms permitting prokaryotic modulation of NF-κB activation following infection in cells outside the gastrointestinal tract is less clear.

The purpose of the present study was: (a) to determine if bacterial signalling from the lumen of the murine gut after feeding with a well characterised commensal/probiotic could impact *in vivo* activation of the pro-inflammatory transcription factor NF-κB following infection thereby limiting infection-associated inflammatory injury; (b) examine the mechanism of such protective regulation by assessing Treg cellular activity in probiotic-fed mice and by adoptive transfer of T cells into naïve non-fed animals. The results show that the putative probiotic/commensal organism drives Treg activity which limits the pro-inflammatory response to invasive salmonella infection via down-regulation of NF-κB activation.

## Results

### Impact of *B. infantis* on the pro-inflammatory response to *S. typhimurium*

**Figure 1:**
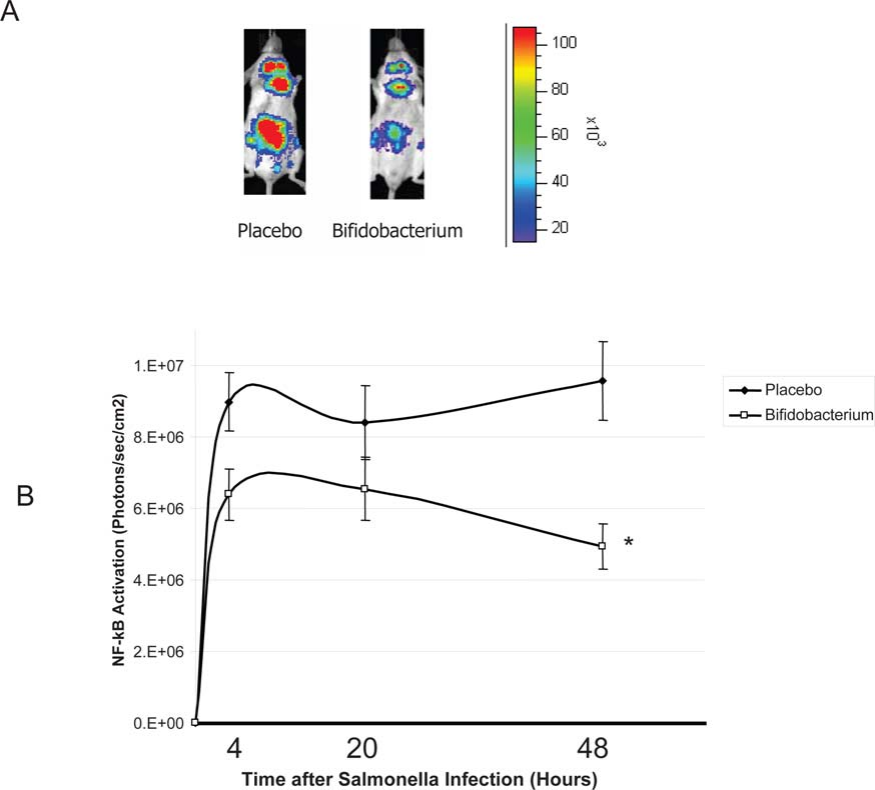
*B. infantis* attenuates NF-κB activation *in vivo* in response to *S. typhimurium* infection. (A) A representative in vivo image illustrates that NF-κB activation in NF-κBlux transgenic mice four hours following *S. typhimurium* infection is attenuated when an animal is pre-fed *B. infantis*; (B) *In vivo* NF-κB activation in *B. infantis*-fed animals was significant reduced compared to NF-κB activation in placebo-fed controls (n = 6/group). *p<0.05 versus placebo.

**Figure 2:**
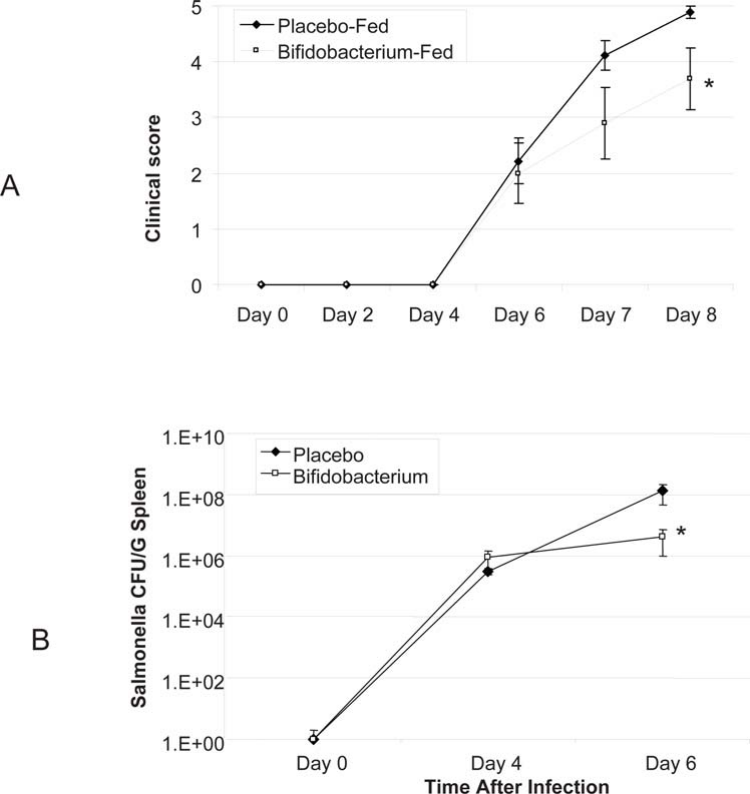
*B. infantis* reduces *S. typhimurium* disease severity and systemic translocation. (A) Macroscopic clinical scoring of mice infected with *S. typhimurium* reveals a significant reduction in *B. infantis* pre-fed mice disease symptoms 8 days following initial infection (n = 10/group); (B) *S. typhimurium* numbers were reduced in the spleens and livers of *B. infantis* fed mice 6 days following infection (n = 18/group). *p<0.05 versus placebo.

Mice consuming *B. infantis* were protected from the effects of infection with *S. typhimurium*. Biophotonic imaging of *in vivo* NF-κB activation illustrated a significant difference just four hours following infection (Figure 1A). *B. infantis*-fed animals exhibited a less pronounced activation of the *in vivo* NF-κB response compared to the NF-κB response to infection in placebo-fed controls. This suppressed pro-inflammatory response persisted for the remainder of the study period (Figure 1B). Murine clinical scores were not significantly different until 7 days after the initial infection at which stage *B. infantis*-fed animals were significantly better that placebo-fed controls (Figure 2A) Similarly, systemic pathogen numbers were not different between the groups until 6 days after infection (Figure 2B). Therefore, *B. infantis* attenuation of NF-κB activation *in vivo* precedes a reduction in *S. typhimurium* disease severity and systemic translocation.

### *B. infantis* reduces the *in vivo* NF-κB response to LPS

**Figure 3:**
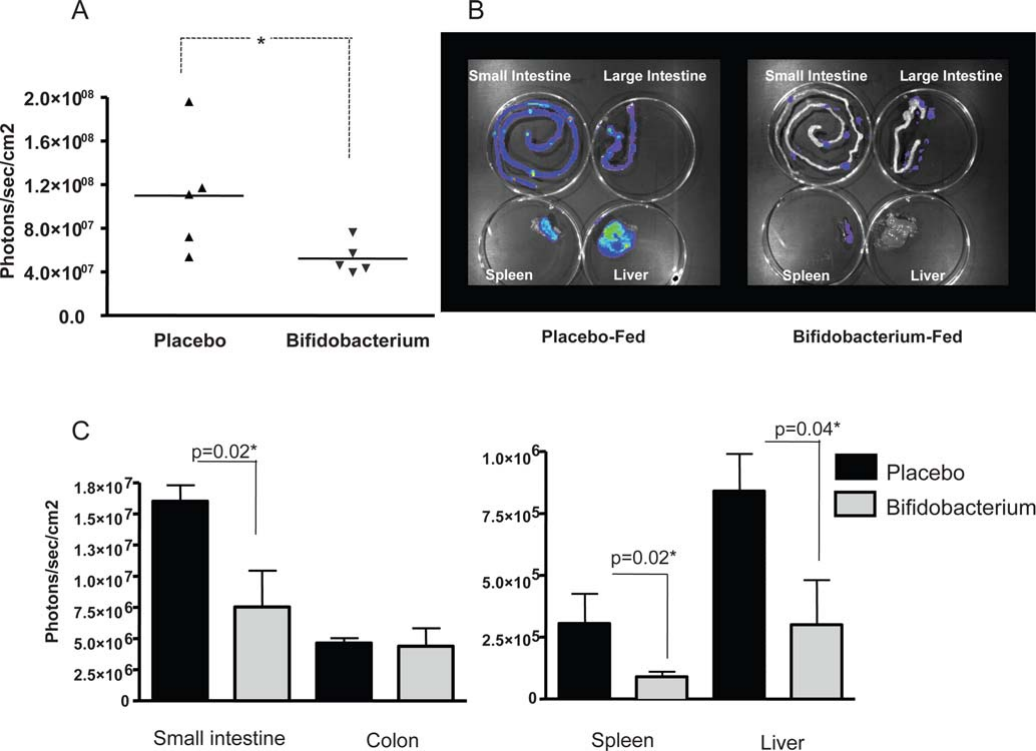
NF-κB activation is suppressed in LPS-injected mice when pre-treated with *B. infantis*. (A) Whole body imaging of NF-κB*lux* mice 4 hours following LPS injection reveals suppressed NF-κB activation in the *B. infantis*-fed animals. (B) Organs were removed for imaging purposes and a representative image is illustrated. (C) The mean increase in NF-κB activation following i.p. injection of LPS is significantly less in the ileum, spleen and liver of *B. infantis*-fed mice. *p<0.05 versus placebo, n = 5/group.

In agreement with the results above suggesting a systemic anti-inflammatory effect of *B. infantis* consumption on *S. typhimurium*-induced inflammatory damage, *in vivo* imaging of the pro-inflammatory transcription factor NF-κB in response to LPS alone demonstrated significantly less activity in mice-fed *B. infantis* four hours after LPS injection (Figure 3A). In order to identify the sites at which NF-κB activity was being suppressed, we removed the small intestine, large intestine, liver and spleen from each animal and quantified NF-kB activity for each organ individually (Figure 3B). NF-kB activation within the small intestine, liver and spleen (but not the colon) was significantly reduced in *B. infantis*-fed animals (Figure 3C).

### *B. infantis* consumption reduces pro-inflammatory cytokine secretion

In order to further characterise the impact of NF-κB suppression on the host inflammatory response, cytokine production by isolated Peyer's patch and spleen-derived cells was assessed, *in vitro*, following anti-CD3/CD28 stimulation (lymphocyte response) or LPS stimulation (innate TLR-4 response) immediately prior to *S. typhimurium* infection (Day 0) or four days (Day 4) after infection.

**Figure 4:**
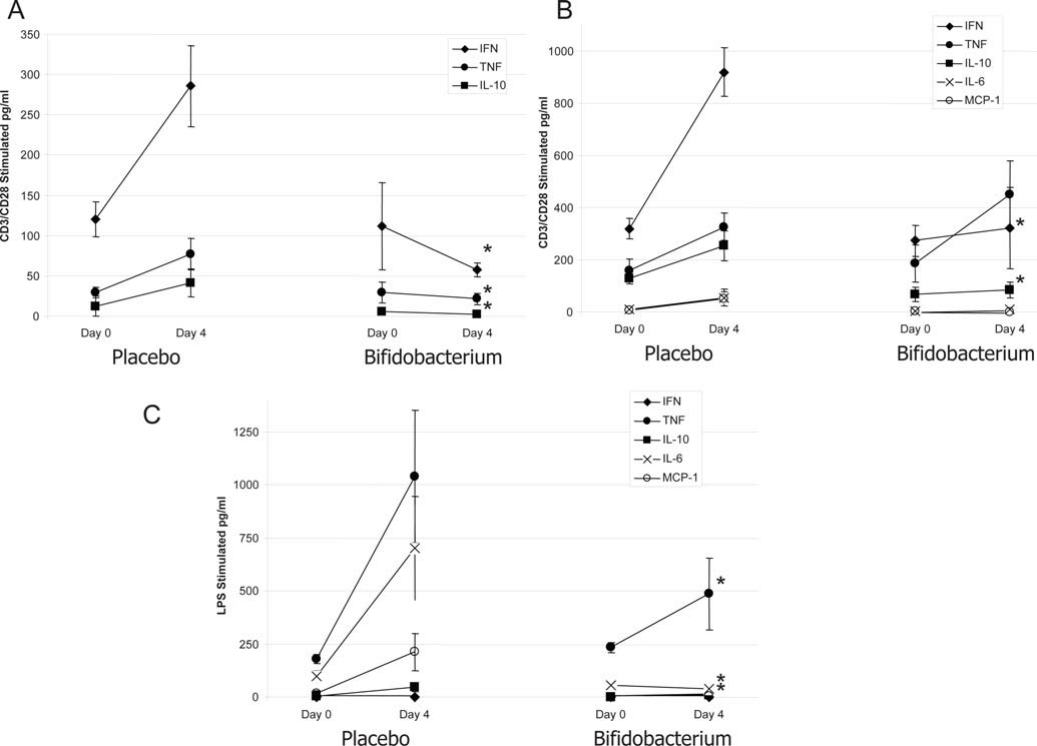
Cytokine production is suppressed in *S. typhimurium*-infected mice when pre-treated with *B. infantis*. Cytokine release by isolated cells was examined immediately prior to *S. typhimurium* infection (Day 0) or four days (Day 4) after infection. (A) *In vitro* cytokine production by anti-CD3/CD28 stimulated Peyer's patch cells is significantly reduced in *S. typhimurium* infected mice when fed *B. infantis* with no differences being observed prior to infection; (B) *In vitro* IFN-γ and IL-10 production by anti-CD3/CD28 stimulated spleenocytes is significantly increased in placebo-fed animals following S. typhimurium infection. However, this increase was not observed in animals consuming *B. infantis*. TNF-α levels were similar for the two groups; (C) LPS stimulated splenocytes released comparable amounts of cytokine from the two groups of animals prior to infection but four days following Salmonella translocation, LPS stimulated TNF-α, IL-6 and MCP-1 release was significantly less in the *B. infantis*-fed animals. *p<0.05 versus placebo, n = 8/group at each timepoint.

CD3/CD28 stimulation of Peyer's patch cells resulted in secretion of IFN-γ, TNF-α and IL-10 with no differences noted between placebo-fed controls or *B. infantis*-fed test animals prior to Salmonella infection (Figure 4A). However, four days of *S. typhimurium* infection was associated with enhanced release of IFN-γ, TNF-α and IL-10 by anti-CD3/CD28 stimulated Peyer's patch cells in placebo-fed animals but not in animals consuming *B. infantis*. *In vitro* stimulation of Peyer's patch cells with LPS yielded cytokine responses below the assay detection limit and were not examined further (results not shown).

CD3/CD28 stimulation of splenocytes resulted in secretion of IFN-γ, TNF-α and IL-10 with low levels of IL-6 and MCP-1 (Figure 4B). In agreement with the Peyer's patch results, no differences in the cytokine secretion patterns were noted for *in vitro* stimulated splenocytes from placebo or *B. infantis* treated mice prior to infection. However, the anti-CD3/CD28 stimulated IFN-γ and IL-10 response was significantly attenuated following Salmonella infection in the *B. infantis*-fed animals. LPS stimulated splenocytes released comparable amounts of cytokine from the two groups of animals prior to infection but four days following Salmonella translocation, LPS stimulated TNF-α, IL-6 and MCP-1 release was significantly less in the *B. infantis*-fed animals (Figure 4C).

### Dendritic cell CD80 expression is altered by *B. infantis* consumption

**Figure 5:**
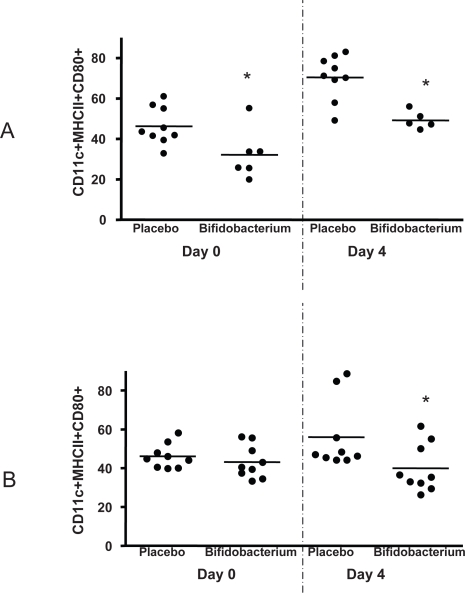
Dendritic cells are less activated in *B. infantis*-fed mice. (A) The percentage of CD11c^+^MHC II^+^ mature dendritic cells staining positive for the co-stimulatory molecule CD80 are reduced in the Peyer's patch of *B. infantis*-fed mice prior to and during *S. typhimurium* infection. (B) Splenocyte dendritic cell CD80 expression is similar in both groups of un-infected mice but is significantly up-regulated with Salmonella infection only in the placebo group. *p<0.05 versus placebo, n = 6–9 animals per group per timepoint.

Presentation of antigen by dendritic cells to naïve T cells requires the expression of co-stimulatory molecules such as CD80 for effective maturation and clonal expansion of the interacting T cells. We examined the percentage of mature dendritic cells (CD11c^+^MHCII^+^) expressing CD80 in both the Peyer's patch and spleen of placebo and *B. infantis*-fed animals. Surprisingly, fewer Peyer's patch dendritic cells co-express CD80 when isolated from *B. infantis*-fed animals (Figure 5A). Dendritic cell CD80 expression increases in the Peyer's patches of all Salmonella infected animals but remains significantly less in the *B. infantis*-fed mice. Splenocyte dendritic cell CD80 expression is similar in both groups of un-infected mice but is significantly up-regulated with Salmonella infection only in the placebo group (Figure 5B).

### *B. infantis* consumption is associated with increased numbers of T regulatory cells

**Figure 6:**
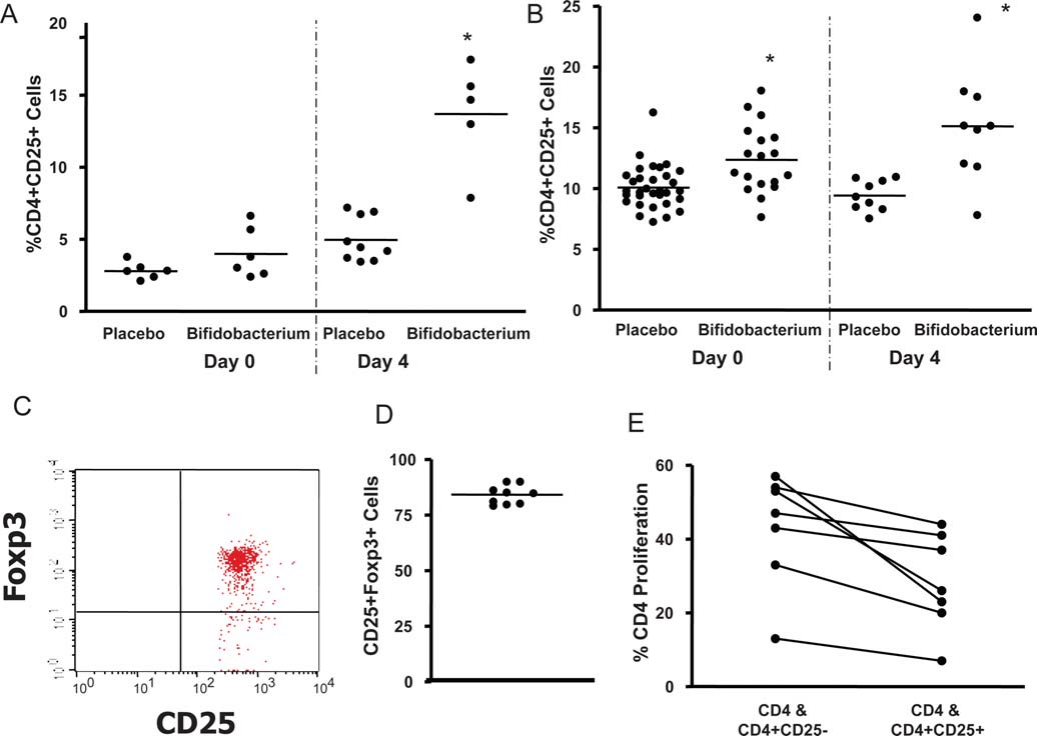
*Bifidobacterium infantis* consumption increases Treg numbers and activity in *S. typhimurium* infected mice. (A) There is a significant increase in the percentage of CD4^+^ cells co-expressing CD25 in the Peyer's patch of animals fed *B. infantis*, particularly following Salmonella infection; (B) There are also significantly more CD4^+^CD25^+^ cells in the spleen of animals fed *B. infantis*; (C) A representative flow cytometry dot-plot illustrates that the majority of the CD25^+^ cells within the spleen also stain positive for Foxp3 which is graphed for *B. infantis*-fed animals in (D); (E) CD4^+^CD25^+^ T cells suppressed proliferation of naïve CFSE labelled CD4^+^ cells while depletion of the CD25^+^ subset cells removed the suppressive effect. *p<0.05 versus placebo.

The percentage of CD4^+^ T cells expressing the α chain of the IL-2 receptor, CD25, was examined in both Peyer's patch and spleen of un-infected and Salmonella infected animals. We noted a significant increase in the percentage of CD4^+^ cells co-expressing CD25 in the Peyer's patch of animals fed *B. infantis*, particularly following Salmonella infection (Figure 6A). A statistical significant inverse correlation was noted between the percentage of CD80^+^ activated dendritic cells and CD4^+^CD25^+^ T cells within the Peyer's patch suggesting a link between dendritic cell co-stimulatory molecule expression and T regulatory cell induction (r^2^ = 0.419, p = 0.01). This does not prove a causal relationship but simply implies that these cellular events may both be associated with bifidobacterial consumption.

There are also significantly more CD4^+^CD25^+^ cells in the spleen of animals fed *B. infantis* (Figure 6B). The majority (>75%) of the spleen-derived CD4^+^CD25^+^ T cells also stained positive for the Treg transcription factor Foxp3 (Figure 6C & 6D). Finally, when these T cells were isolated and examined *in vitro*, the CD4^+^CD25^+^ T cells suppressed proliferation of naïve CFSE labelled CD4 cells while depletion of the CD25^+^ subset cells removed the suppressive effect (Figure 6E).

### CD4+CD25+ T cell adoptive transfer reduces *in vivo* NF-κB activation

**Figure 7:**
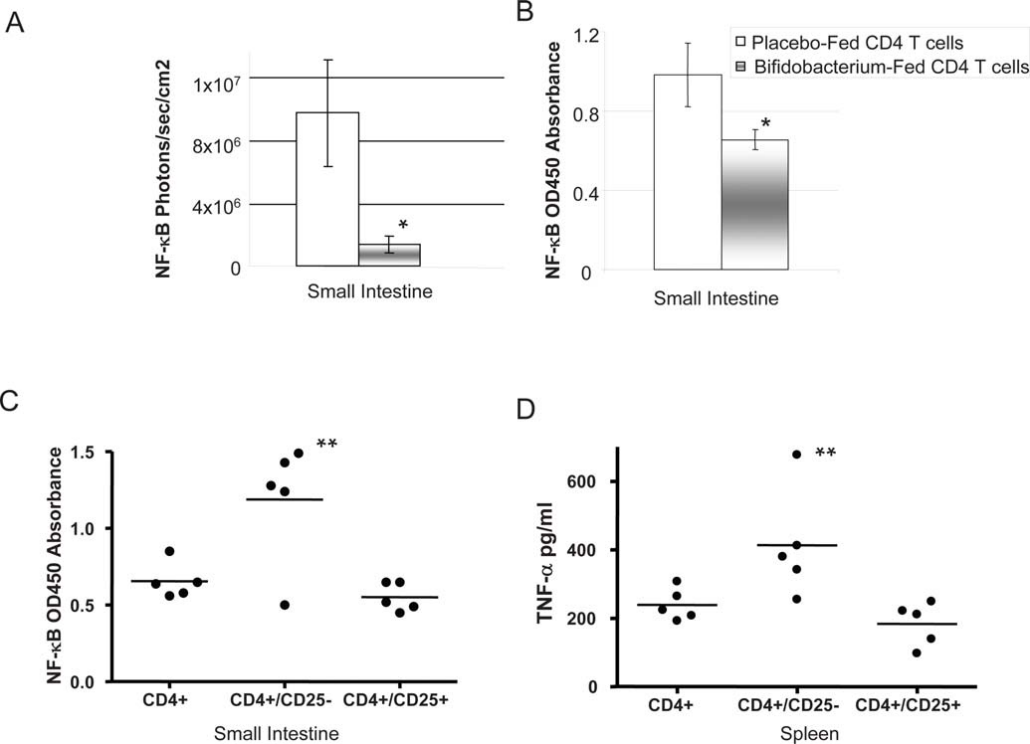
NF-κB activation & TNF-α secretion in response to LPS is significantly reduced by adoptive transfer of CD4^+^CD25^+^ T cells from *B. infantis*-fed animals. (A) In vivo adoptive transfer of CD4^+^ NF-κB^−/−^ T cells from *B. infantis*-fed animals resulted in significant attenuation of NF-κB activity following i.p. LPS administration in NF-κBlux^+/+^ animals compared to mice that received T cells from placebo-fed controls (n = 6/group); (B) A subsequent study performed an identical adoptive transfer experiment except that nuclear NF-κB activation was measured using an ELISA system instead of biophotonic imaging - identical results were observed in that CD4^+^ T cells from *B. infantis*-fed animals significantly reduced LPS stimulated NF-κB activation (n = 5/group); (C) Removal of the CD25^+^ subpopulation from the CD4^+^ cells resulted in loss of the NF-kB suppressive activity while adoptive transfer of the CD25^+^ population alone replicated the CD4^+^ suppressive effect; (D) Similarily, the reduction in TNF-α secretion associated with CD4^+^ T cell transfer was lost when CD25^+^ were depleted but was replicated with transfer of CD25^+^ cells alone (n = 5/group). *p<0.05 versus placebo T cells; **p<0.05 versus CD4 T cells.

In order to examine the hypothesis that NF-κB suppression is mediated by bifidobacterial induction of Tregs, we isolated CD4^+^ T cells from placebo or *B. infantis*-fed animals and adoptively transferred these to naïve animals. Following LPS injection, NF-κB activation was significantly less in animals that received CD4^+^ T cells from *B. infantis*-fed mice compared to animals that received CD4^+^ T cells from placebo-fed mice (Figure 7A & 7B). Depletion of the CD25^+^ subset from the T cell population removed the suppressive effect while adoptive transfer of CD25^+^ cells alone was sufficient to reduce NF-κB activation *in situ* and release of TNF-α from cultured splenocytes (Figure 7C & 7D).

## Discussion

This report illustrates at a cellular and molecular level the impact of the commensal microbiota on host immune defence and immune homeostasis. The deliberate consumption of one commensal organism, *Bifidobacterium infantis* 35624, resulted in the induction of Treg cells which protected the host from excessive inflammation during the course of infection as evidenced by reduced pro-inflammatory cytokine production, reduced T cell proliferation, reduced dendritic cell co-stimulatory molecule expression and attenuation of NF-κB activation. The role of Treg cells in this biological process was conclusively demonstrated by the adoptive transfer of CD4^+^CD25^+^ T cells into naïve mice which was sufficient to suppress NF-κB activation in response to LPS injection.

Treg cells have been well described as suppressors of auto-reactive T cells [14],[15] and suppress inflammatory disease in a wide range of murine models including experimental autoimmune encephalomyelitis [16], inflammatory bowel disease [17], bacterial-induced colitis [18], collagen-induced arthritis [19], type I diabetes [20], airway eosinophilic inflammation [21], graft-vs-host disease [22] and organ transplantation [23]. Similarly, supplementation of the microbiota with certain commensal micro-organisms has also shown anti-inflammatory effects in a number of these models [24]–[27]. It is tempting to speculate that induction of Treg cells by the commensal microbiota could be partly responsible for the observed anti-inflammatory activity in these model systems. However, the role of commensal bacteria in controlling the pro-inflammatory response associated with infection is less clear. Indeed, the activity of Treg cells in the models above is primarily mediated by suppression of antigen specific immune responses while our studies demonstrate an effect on innate immune activation, in particular activation through the TLR-4 pathway. It has been recently suggested that CD4^+^CD25^+^ T cells induce an alternative activation pathway in monocytes associated with a diminished capacity to respond to LPS which supports the findings of this study [28],[29].

The host cellular mechanisms underpinning induction of Treg cells by the commensal microbiota was not examined in this study but is hypothesised to be mediated in part by dendritic cells. Following encounter with pathogenic or commensal microbes, dendritic cells provide instructive signals that influence T cell differentiation into Th1, Th2 or regulatory phenotypes [30],[31]. Multiple reports implicate dendritic cells in the induction of tolerance and regulatory cells to the commensal microbiota [32]. *In vitro* co-incubation of human mesenteric lymph node derived dendritic cells with *B. infantis* resulted in the secretion of IL-10 and TGF-β, but not TNF-α or IL-12 [33]. Both IL-10 and TGF-β are important cytokines in directing naïve T cell maturation down a regulatory pathway. In addition, *in vitro* co-incubation of monocyte derived dendritic cells with certain commensal lactobacilli strains have been shown to drive the *in vitro* generation of IL-10 producing T cells which inhibit bystander T cell proliferation [34].

Not all commensal micro-organisms may be equally effective in driving T regulatory cell activity. Co-incubation of a panel of commensal bacteria with peripheral blood mononuclear cells (PBMCs) *in vitro* resulted in varying amounts of IL-10 production suggesting that strain specific characteristics, as yet largely undefined, drive regulatory cytokine production [35]. Commensal-induced protection from infection may also involve additional strain-specific mechanisms of action including the production of selective anti-microbial compounds [2]. The relative importance of microbe-microbe and microbe-host interactions in protecting the host from infection is likely to also depend on the nature of the infectious organism. However, the mechanism underpinning the suppressed pro-inflammatory response to a translocating microbe described in this study strongly implicate Tregs as modulation of the host pro-inflammatory response precedes the difference in systemic salmonella recovery. In addition, LPS injection models systemic infection with a gram negative pathogen and suppression of the host response to LPS alone can not be explained by other mechanisms such as competition within the intestine or activation of pathogen-specific effector T cells.

This report clearly demonstrates that activation of the host innate pro-inflammatory pathways to a translocating infectious agent, or systemic LPS, can be influenced by the commensal microbiota via the induction of Treg cells. NF-κB is a key transcription factor that is central to the observed anti-inflammatory effect and improved regulation of NF-κB is an important therapeutic target in a wide range of pro-inflammatory states, including sepsis [36]. This report supports the clinical evaluation of appropriately selected probiotic/commensal micro-organisms for the promotion of CD4^+^CD25^+^Foxp3^+^ T cells in vivo in order to control the innate inflammatory cascade to translocating microbes.

## Materials and Methods

### Animal models

Balb/c mice were obtained from Charles River Laboratories (Bicester, UK) and bred in-house for bifidobacterial feeding and salmonella infection studies. NF-*k*B*lux* transgenic mice on a C57BL/6J-CBA/J background were obtained from Charles River Laboratories (Wilmington, USA) and bred in-house. Mice were housed under barrier maintained conditions within the biological services unit, University College Cork (UCC). All animal experiments were approved by the UCC animal ethics committee and experimental procedures were conducted under appropriate license from the Irish government.

### Bacterial strains

*Bifidobacterium infantis* 35624 (*B. infantis*) was isolated from healthy human gastrointestinal tissue and its use as a probiotic organism has been previously reported [37],[38]. *B. infantis* was routinely cultured anaerobically for 48 hours in deMann, Rogosa and Sharpe medium, MRS, (Oxoid, Basingstoke, UK) supplemented with 0.05% cysteine (Sigma, Dublin, Ireland). *Salmonella typhimurium* UK1 (*S. typhimurium*) was provided by Roy Curtis (Washington University, US) and was routinely cultured aerobically at 37°C for 24 hours in tryptic soya broth (Oxoid).

### Animal model feeding studies

*B. infantis* was administered to all animals as a freeze-dried powder reconstituted in water at approximately 1×10^9^ colony forming units/day/animal. Mice consumed the commensal micro-organism in their drinking water *ad libitum* for at least 3 weeks prior to salmonella infection. Challenge with *S. typhimurium* was performed as described by Wilems-Riesenberg *et al* [39],[40]. Briefly, *S. typhimurium* was grown overnight, pelleted and resuspended in buffered saline gelatin (0.85% NaCl, 0.01% gelatin and 2.2 mM KH_2_PO_4_). Inoculation with Salmonella was performed by injecting 20 µl of a single inoculum of the Salmonella suspension behind the murine incisors with a micropipette tip in a flexible film isolator. A dose of 1×10^6^ *S. typhimurium* viable cells was chosen as the optimal dose for these studies as higher doses did not result in increased recovery of viable cells from the murine liver and spleen while lower doses did not yield reproducible or consistent bacterial counts from systemic sites [41]. Animals were monitored for disease progression using a clinical scoring scale. This scale scored animals from 0 (no disease) to 7 (severe disease) and is illustrated in Table 1.

**Table 1:** Disease Severity Scale.

Stool	Blood in stool	Appearance
0. Well formed pellets	0.No blood	Fur texture:
1. Changed formed pellets	1.Blood	0. Smooth, 1. Scruffy
2. Loose stool	2.Gross bleeding	Anal prolapse:
3. Diarrhea/No stool		0. Not present, 1. present

Salmonella titres were calculated in liver and spleen using quantitative real-time Polymerase Chain Reaction (qPCR). Briefly, liver and spleen samples were transferred to sterile stomacher bags and mixed with 1X PBS (Gibco BRL, UK) and homogenised using mechanical means. Samples were stored at-20°C until analysis. DNA extractions were performed using the Qiagen DNAeasy Tissue Kit (Qiagen,West Sussex, UK) according to manufacturers instructions. Isolated DNA concentrations were ascertained using the Nanodrop (Thermo Scientific) and standardised to 20 ng DNA per reaction. LightCycler Real-time PCR was used to quantify Salmonella genomic DNA concentrations using the following primers INVA2_R: 5′-TGT CCT CCG CTC TGT CTA CTT -3′ INVA2_L: 5′-ATC AAC AAT GCG GGG ATC T -3′ (76 bp product), and Probe library #9 (Roche Diagnostics). All primers were synthesized by MWG Biotech, (Ebersberg Germany). PCR amplifications were carried out in glass capillaries (Roche Diagnostics) in a total final volume of 20 µl using a LightCycler (Roche Diagnostics). A standard curve was generated by adding DNA from 10 fold dilution series of purified Salmonella genomic DNA. This was used to calculate Salmonella concentration. The LightCycler software identified the Ct (threshold cycle number) values and the concentrations of Salmonella were calculated by comparing Ct values to the crossing point values of the linear regression line of the standard curve.

### *In vivo* assessment of NF-κB signalling

*In vivo* imaging of NF-*k*B*lux* transgenic mice were performed by firstly anaesthetizing the mice with isoflurane (Inhalation Anaesthetic, Abbot Laboratories Ltd., Kent, UK). D-luciferin (120 mg/kg; Biothema AB, Handen, Sweden) dissolved in 200 *ul* PBS, pH 7.8, was injected i.p. Immediately afterwards the mice were placed in a ventral recumbent position in a light-sealed chamber in the *In Vivo* Imaging System (IVIS) chamber (Xenogen, Alameda, USA) and imaged continuously for 5 minutes with a medium sensitivity setting starting 2 minutes after the injection of D-luciferin. A reference black and white image of the animal was taken in low light conditions then a sensitive cooled charge-coupled device camera collected the photons emitted. Photons were quantified using Living Image software (Xenogen) and the luciferase activity quantified as the amount of light emitted per second per cm^2^ from the animal/organs. The pseudo-colored images represent light intensity (red is the strongest and violet is the weakest).

Individual organs to be imaged were excised from the mice 5 minutes following D-luciferin administration. Organs were placed in a culture dish and immediately imaged, again with an acquisition integration time of 5 minutes. Organs examined were the spleen, liver, small intestine and colon.

### *In vitro* culture and cytokine measurement

Spleens were aseptically removed from all animals and a single cell suspension generated using mechanical means. In addition, the small intestine of mice were removed and the lymphoid follicles of the Peyer's patches were carefully dissected from the intestinal serosal side with curved scissors and collected into 5 ml of PBS containing 1 mM EDTA and collagenase (Sigma). Following incubation in a shaking oven at 37°C for 20 mins, the collected patches were placed between two sterile glass slides and crushed. This cell suspension was centrifuged (100 g×10 min) and the pellet was resuspended and diluted in Dulbecco's modified eagle medium (DMEM). Single cell suspensions were seeded, in duplicate, in 24 well tissue culture plates (Sarstedt, Newton, USA) at 1×10^6^ cells per well. Single cell suspensions were stimulated for 72 hours with anti-CD3 and anti-CD28 antibodies (BD Biosciences, Oxford, UK), LPS (Sigma) or remained non-stimulated to assess background cytokine secretion. Following a 72-hour incubation period (@37°C and 5% CO_2_ humidified atmosphere) all supernatants were harvested for cytokine analysis. These were aliquoted and stored at −70°C for analysis of cytokine production in batches. IL-6, IL-10, IL-12, MCP-1, TNF-α and IFN-γ levels were quantified using cytometric bead arrays (BD). Cytokine levels were measured using a BD FacsCaliber flow cytometer and analysis was carried out using the BD CellQuest software and BD CBA Software.

### Proliferation assay

CD4 T cell proliferation was measured using the CellTrace™ CFSE Cell Proliferation Kit (Invitrogen). Briefly, CD4^+^ T Cells were positively selected (>98% purity) from spleens using CD4 MicroBeads (Miltenyi Biotech, Bergisch Gladbach, Germany) and an autoMACS separator. These cells were labeled with CFSE and incubated in vitro for three days with anti-CD3 and anti-CD28 stimulation (BD). Concurrently, CD4^+^ T cells were isolated from B. infantis-fed mice as outlined above and subdivided into CD4^+^CD25^+^ and CD4^+^CD25^−^ populations using microbeads (Miltenyi). These cells were not CFSE labeled and were co-incubated with the labeled cells for the three day stimulation period. Lymphocyte proliferation was measured by flow cytometry.

### Cellular phenotypes

Single cell suspensions from the Peyer's patch and spleens of mice were generated as outlined above. Monoclonal antibodies to CD3, CD4, CD11c, CD25, CD80 and MHC II (BD) were used to label cells for T cell subset analysis (CD3, CD4 and CD25) and dendritic cell co-stimulatory molecule expression (CD11c, MHC II and CD80). Antibodies to the transcription factor Foxp3 (eBioscience, San Diego, USA) were used to label permeabilised cells in representative experiments. Cellular phenotypes were measured using a BD FacsCaliber flow cytometer and analysis was carried out using the BD CellQuest software.

### Impact of CD4^+^CD25^+^ T cell adoptive transfer on NF-κB activation

*In vivo* assessment of NF-κB activation was determined following feeding of wildtype (NFkB^−/−^) mice with either *B. infantis* or placebo for three weeks. The spleens were removed and CD4^+^ T Cells were positively selected (>98% purity) using the method outlined above. Adoptive transfer of 1×10^6^ CD4^+^ T cells/ml into NF-*k*B*lux* ^+/+^ transgenic mice (n = 6/grp) was performed by i.p. injection. 8 days later 3 mg/kg LPS (Sigma) was injected i.p. and NF-κB measurements taken after 4 hours. *In vivo* images were captured by the IVIS in the same manner as described above.

In order to determine the CD4^+^ T cell subset responsible for mediating this effect, CD4 T cells from *B. infantis*-fed animals were further isolated into CD4^+^/CD25^+^ and CD4^+^/CD25^−^ subpopulations as per manufactures protocol (Miltenyi Biotech, Bergisch Gladbach, Germany). Adoptive transfer of 1.5×10^5^ CD4^+^/CD25^+^ T cells/ml or 8.5×10^5^ CD4^+^/CD25^−^ T cells/ml into naïve mice (n = 5/grp) was performed by i.p. injection. 8 days later 3 mg/kg LPS (Sigma) was injected i.p. and animals were sacrificed after 4 hours and tissues harvested. Intestinal NF-κB p65 activation was measured in nuclear extracts using an NF-κB p65 ELISA-based transcription factor assay kit (TransAM Assay, Active Motif, Germany) according to the manufacturer's protocol. TNF-α release from LPS stimulated splenocytes was measured using flow cytometry as outlined above.

### Statistical analysis

GraphPad Prism software utilising 2Way-ANOVA with Bonferroni's Post-test was used to determine statistical significance. LightCycler data was analyzed using Pearson's correlation coefficient and students T-tests were used to determine differences between groups.
